# Positive effects of lumbar puncture simulation training for medical students in clinical practice

**DOI:** 10.1186/s12909-020-02452-3

**Published:** 2021-01-06

**Authors:** Sinead Gaubert, Alice Blet, Fadia Dib, Pierre-François Ceccaldi, Thomas Brock, Maude Calixte, Léa De Macédo, Tiphaine Dujardin, Ludivine Jean-Louis, Dhihia Leghima, Samuel Mouyal, Dan David Tordjman, Patrick Plaisance, Caroline Roos, Sid-Ahmed Remini, Damien Roux, Claire Paquet

**Affiliations:** 1 Université de Paris, Medical School, Paris, France; 2 Cognitive Neurology Center, AP-HP, Lariboisière Fernand-Widal Hospital, F-75010 Paris, France; 3 Surgical intensive care unit, AP-HP, Lariboisière Fernand-Widal Hospital, F-75010 Paris, France; 4 INSERM CIC 1417, F-CRIN, I REIVAC, AP-HP, Hôpital Cochin, F-75014 Paris, France; 5 INSERM, Sorbonne Université, Institut Pierre Louis d’Épidémiologie et de Santé Publique, Paris, France; 6 Department of Gynecology and Obstetrics, AP-HP, Beaujon-Bichat Hospital, F-92110 Clichy, France; 7 Emergency Unit, AP-HP, Lariboisière Fernand-Widal Hospital, F-75010 Paris, France; 8 Cephalalgia Center, AP-HP, Lariboisière Fernand-Widal Hospital, F-75010 Paris, France; 9 Intensive Care Unit, AP-HP, Louis Mourier Hospital, F-92700 Colombes, France

**Keywords:** Lumbar puncture, Simulation training, Clinical skills, Medical education, Medical student

## Abstract

**Background:**

Lumbar puncture (LP) is an invasive medical procedure that can be done by any doctor. Several simulation-based trainings have been built however the evaluations of the theoretical knowledge and the impact of the simulation-based training have never been performed in real life.

The objective was to evaluate the impact of a LP training on the theoretical knowledge improvement and the performance of a LP in clinical practice.

**Methods:**

Before and after medical students’ training, theoretical knowledge and confidence level were assessed. Over a 6 months period, the impact of simulation training was evaluated by the success rate of students’ first LP carried out in hospitalized patients and compared to the results of a no-training control.

**Results:**

Students’ theoretical knowledge and confidence level showed significant improvement after simulation training on 115 students (*p* < 0.0001). The evaluation in real life based on 41 students showed that the success rate of the first LP in patients was higher in the LP simulation group compared to the control group (67% vs 14%, *p* = 0.0025). The technical assistance was also less frequently needed in the LP simulation group (19% vs 57%, respectively, *p* = 0.017). The rate of students who participated in this educational study was low.

**Discussion:**

Simulation-based teaching was an effective way to improve students’ theoretical and practical knowledge. Whether this approach translates to other procedural skills in real clinical settings merits further study. The low participation rate in the study is due to the fact that students are not used to be included in educational studies and to the complexity of evaluation in routine clinical practice.

**Supplementary Information:**

The online version contains supplementary material available at 10.1186/s12909-020-02452-3.

## Background

Lumbar puncture (LP) is a common invasive medical procedure performed by many physicians from various specialties. It can cause side effects which could be avoided by a good knowledge and proper practice of the procedure [1–4]. Several studies have demonstrated a high variability in LP practice leading to variable rate of side effects [1, 3], reflecting a lack of good knowledge.

Healthcare simulation is an “educational method that replaces or amplifies real experiences with guided experiences replicating substantial aspects of the real world in a fully interactive manner”. Simulation allows progression of skills from novice to expert in a safe environment for the learner using a simulated patient.

Previously, few studies have demonstrated that the use of simulation-based LP training is more effective than the “traditional” clinical training where students learn LP directly in clinical practice with a patient [5–12]. In these studies the efficacy of simulation training was evaluated on the simulator itself and not in real clinical practice in the ward [6, 7, 10, 11, 13]. Meanwhile, we have noted a lack of theoretical teaching and official recommendations for LP practice.

Taking into account the lack of theoretical teaching and evaluation and the lack of evaluation in real patients in the education literature, we set up a specific training including both theoretical and practical sessions. In this study our objectives were to evaluate 1/ the impact of LP simulation training on the level of theoretical knowledge, 2/ the rate of success in real clinical conditions using a randomized study.

## Methods

### Participants

In French Medical Education, medical students begin to perform medical procedures in patients during the fifth-year level. Then, our study included two groups of fifth-year medical students. In a first group of medical students, between January 2015 and September 2017, we evaluated the impact of theoretical teaching and LP simulation training on the LP knowledge and confidence in the performance of LP in real practice. In a second group of medical students, during 2017–2018 academic year, we conducted a prospective randomized study comparing the rate of LP success in real life in students exposed or not to prior LP simulation training.

### Evaluation methods

Concerning the assessment of theoretical knowledge, the evaluation was based on Multiple Response Questions (MRQ) results before and after training. In the randomized study, evaluation was based on the successful rate of the first LP performed by students. To ensure the feasibility, we have chosen to evaluate only the first LP performed by the student.

### Evaluation of LP simulation training on theoretical knowledge

Between January 2015 and September 2017, we trained 115 medical students from Université de Paris. As a first step, we delivered to students a theoretical teaching by an e-mail document about LP prior to the live simulation-based training session based on the review of the literature [2].

All sessions were conducted in groups of six students on LP simulators from Kyoto Kagaku. The design of the LP simulation training session included the following sections: 1- At the beginning of the session, students’ theoretical knowledge was assessed by MRQs (Supplementary file 1A). 2- Then, students had to indicate in a survey their self-assessed level of theoretical knowledge and confidence in performing LP by an auto-evaluation using a scale from 0 to 10 giving a mark out of 10 (pre-test part) (supplementary file 1B). 3- Students had to complete the same survey a second time at the end of the simulation training session (post-test part). 4- Finally, students were asked to complete a satisfaction survey regarding the quality of the training session.

The training session’s theoretical objectives included the following items: 1) list the most frequent side effects and contraindications of LP; 2) list the precautions to be taken to prevent post-LP headache (PLPH); 3) diagnose and treat PLPH. The practical objective was to learn how to perform LP on the mannequin in sitting and lying positions.

### Prospective randomized study on medical students

To assess the impact of LP simulation-based training in real clinical practice, we conducted a monocentric prospective controlled simple-blind randomized study comparing students who underwent or not LP simulation training. All students were informed of the study and of the possibility to refuse to participate. Exclusion criteria were: students who had already received LP simulation-based training or who had previously performed a LP on a patient.

Fifth-year medical students were randomized at the beginning of the 2017–2018 academic year into two groups: an experimental “simulation” group receiving LP theoretical teaching (document sent by e-mail prior to the simulation session) and LP simulation training, and a control group receiving only traditional “clinical training” at the hospital. The first LP in a patient had to be done within a six-month period, in the presence of a physician and, in a training Hospital affiliated with the Université de Paris (France).

Our two main endpoints were 1/ the need for technical assistance given by the supervisor. Technical assistance was defined by the fact that the supervisor would touch the needle to help the student performing the LP, 2/ the successful collection of cerebro-spinal fluid (CSF), without any technical assistance from a supervisor. The student had to collect enough CSF to allow a complete analysis (at least three 1 ml-tubes). Only the first LP was evaluated in participating students. The other endpoints were: 1) the number of puncture attempts; 2) the students’ subjective experience for the first LP in a patient assessed by an electronic survey completed just after the LP, under the control of the supervisor.

### Statistical analyses

We performed group comparisons using Mann–Whitney test or Fisher’s exact test on GraphPad Prism version 5.00 (GraphPad Software, California USA). *p* < 0.05 was considered significant.

## Results

### Evaluation of theoretical knowledge after LP simulation training

**Fig. 1:**
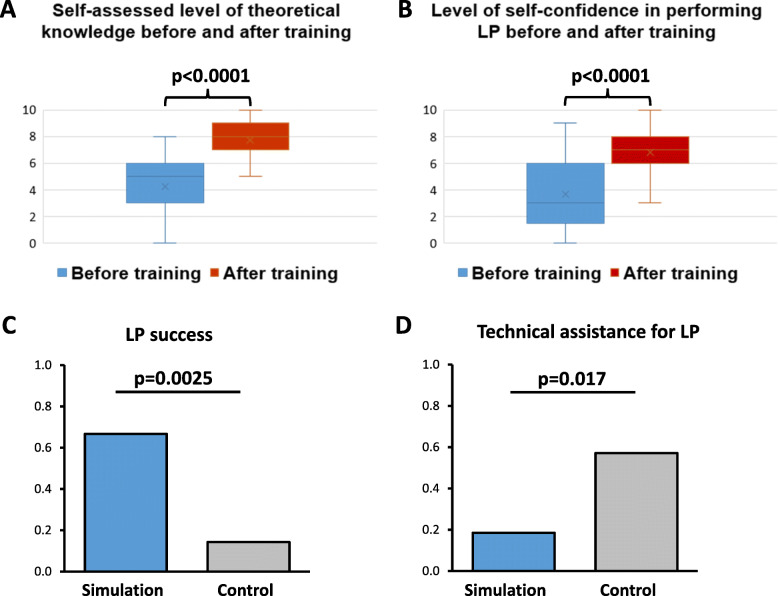
**a** Self-assessed level of theoretical knowledge before and after training. **b** Level of self-confidence in performing LP before and after training. **c** Comparison of success rate of first LP between groups of students with or without prior training. **d** Comparison of need of technical assistance for first LP between groups of students with or without prior training. In (**a**) and (**b**): Box plot displaying the minimum, the quartiles (Q1, Median, Q3), and the maximum values of the data (mark out of 10). Crosses indicate the mean value. *P* value was calculated with Mann–Whitney test. In (**c**) and (**d**): Column bar graph displaying the mean value of the data. P value was calculated with Fisher’s exact test

One hundred fifteen medical students were trained and completed the surveys before and after the LP simulation training sessions carried out at Université de Paris. Each student participated in one training session (2 h). Students’ MRQ mean score was 0.44/1 (SD 0.14), showing a low level of theoretical knowledge prior to the training session. Post-test self-assessments were significantly improved compared to pre-tests, for theoretical knowledge (mean score out of 10: 7.7 [SD 1.2] versus 4.3 [SD 1.9], respectively, *p* < 0.0001), and for confidence level in performing LP (mean score out of 10: 6.8 [SD 1.4] versus 3.7 [SD 2.5], respectively, *p* < 0.0001) (Fig. 1a and b). The level of satisfaction in students was very high, with a mean satisfaction score of 3.8/4 (SD 0.14) (Supplementay file 1C).

### Prospective randomized study on medical students

During the 2017–2018 academic year, all fifth-year medical students (*n* = 388) were randomized into two groups, with 194 students in each group (simulation group and control group). No student refused to participate. Students from the simulation group (*n* = 194) underwent the training session in October 2017. Between October 2017 and May 2018, 41 students (10.6%) performed a first LP in a patient and filled in the electronic survey. There were 27 students (66%) in the simulation group and 14 students (34%) in the control group (Supplementary file 1D). The success rate of the first LP on a patient was significantly higher in the simulation group compared to the control group (67% versus 14%, respectively, *p* = 0.003) (Fig. 1c). Technical assistance from a supervisor was significantly less frequent in the simulation group compared to the control group (19% versus 57%, respectively, *p* = 0.017) (Fig. 1d). No difference between groups was observed neither regarding students’ subjective experience nor the number of puncture attempts.

## Discussion

In this study, we showed that 1/LP simulation training was an appropriate way to improve students’ theoretical knowledge and confidence level in performing LP, 2/ LP simulation training improved the success rate and the autonomy of the students’ in performing LP as they required less technical help in real life.

To our knowledge, the theoretical part of technical procedures (pleural puncture, biopsies …) is sparsely taught. Furthermore, Cranach et al. have demonstrated that LP was associated with the lowest baseline levels of experience and confidence compared to other procedures. This lack of proper teaching leads to heterogeneous practices associated with a variable rate of side effects [1, 3, 14]. In this study, we have demonstrated that teaching with simulation-based sessions is a good opportunity to improve the theoretical knowledge and the technical skills of LP procedure and consequently to optimize LP practice at the hospital. In addition, following this study and in order to improve the theoretical knowledge for students and medical doctors, we have written official national recommendations for the French High Authority of Health (HAS) [15]. We could assume that a similar approach is put in place with other medical procedures in which good knowledge is required to optimize proper practices. Based on these results, we can recommend that theoretical teaching be included in all simulation-based sessions for technical procedures. At the same time, the evaluation of the efficiency of LP simulation training in clinical practice has been poorly explored. Using the evaluation on the simulator itself, several studies have demonstrated the improvement of the students’ skills [6, 7, 10, 11]. Three studies have addressed the question of the impact of LP simulation training in real clinical conditions [5, 9, 12]. One randomized study has evaluated the impact of LP simulation training on the self-reported clinical success of the first LP in infant with 17 trained students and 15 controls. They have demonstrated higher rates of clinical success in the trained group [9]. In a randomized study, Sun and Qi have shown that method-problem and simulator-based learning improve performance based on the evaluation of 10 LP, while they didn’t find any difference between the two groups just after the training [12]. They conclude that the improvement of non-technical skills is needed to improve the performance of LP practice. More recently, Lydon et al. have retrospectively compared the number of successful LP and the number of traumatic LP between two groups and have found a significant difference between trained and non-trained groups [5]. In keeping with our findings, all those studies have demonstrated the improvement of the confidence of the student in LP practice after simulation-based training. They have also shown an impact on LP realization in clinical practice. However, those 3 studies had common limits: 1/the lack of theoretical evaluation, 2/ the absence of information about the number of attempts before succeeding LP, 3/ the lack of assessments concerning the technical or oral help during the LP, 4/ the heterogeneity of the group with various experience of LP.

In our study, we have chosen younger students to ensure the absence of previously performed LP. All students have worked and filled in questionnaires under the supervision of a physician. We have considered students’ success only for the first LP attempt. The increased success rate and significant reduction in the need for technical help during the procedure demonstrates the autonomy of the students performing the LP and this finding is concordant with the evaluation of Sun and Qi in 10 LP. The main limit of our study is the low rate of participation due to the fact that students are not used to be included in educational studies and due to the complexity of evaluation in routine clinical practice (first LP, presence of a referent supervisor who knows the study and the LP recommendations). These methods have reduced the evaluation to only the first LP and the number of available clinical departments with a trained supervisor have probably led to a low rate of participation. Furthermore, students who were involved were still young and not yet affected full time to a specific hospital limiting the possibility of participation. However, the sample size allowed us to demonstrate a significant difference in the rate of success in performing LP.

Overall, those studies have demonstrated the value for medical students of LP simulation-based learning for their level of autonomy (need of help), their self confidence in the procedure and the rate of success. Our study has emphasized the need for theoretical teaching and the need to involve students in educational research studies to improve future clinical practice. Considering the impact of simulation based-training in various specialties, based on several meta-analyses we observed that the evaluation of simulation based-training in real life is poorly explored. Most of the studies limit their evaluation to a simulated environment. Furthermore, the impact and efficiency of simulation-based training is variable according to the studies [16, 17]. All those findings highlight the difficulties performing comparable studies that include real life situations. In this context, our method of real-life evaluation is new. Interestingly, in a meta analyze, Huang et al. 2019 have concluded that the long-time retention of benefits is controversial, and suggest that these benefits may not be transferred to the real-life situations [18]. These results are not in line with our study.

## Conclusion

In this study, we have demonstrated that simulated-based LP training is efficient to improve knowledge and skills for real life LP procedure. We have highlighted that the evaluation of simulated-based training in real life is feasible but difficult, which explains, at least in part, the limited number of studies carried out using this methodology. The measure of its impact in real life is essential in order to adapt and improve our teaching. Methodology to measure the impact of this teaching should be extensively organized in all specialties.

## Supplementary Information

**Additional file 1.**

## Data Availability

Data is available upon request.

## Abbreviations

LP: Lumbar puncture
MRQ: Multiple Response Questionnaire
CSF: Cerebro-spinal fluid
PLPH: Post-lumbar puncture headache
SD: Standard deviation
