# Supplementary material for: Positive effects of lumbar puncture simulation training for medical students in clinical practice
Source: BMC Med Educ. 2021 Jan 6;21:18. doi: 10.1186/s12909-020-02452-3 (PMC7789333; doi:10.1186/s12909-020-02452-3)
Supplement: Supplementary file 1 — Additional file 1. [file 12909_2020_2452_MOESM1_ESM.docx]

***Supplementary file 1***

***A/ Multiple Response Questions***

**MRQ1:** The risk of developing post lumbar puncture headache is

- Depending on the quantity of cerebrospinal fluid withdrawn
- Depending on the diameter of the needle
- Depending on the position of the patient during the lumbar puncture
- Increased with age
- Depending on the kind of needle

**MRQ2:** Prevention of post-lumbar puncture syndrome?

- Bed rest for 24 hours
- Bed rest for several hours
- Use a small diameter needle
- Use an atraumatic needle
- Use a larger diameter needle that decreases the duration of the LP

**MRQ3:** What are the indications for emergency lumbar puncture?

- Meningitis
- In case of unusual headache
- To determine the cause of a progressive motor deficit
- In case of acute headache
- In case of suspicion of Guillain-Barre Syndrome

**MRQ4:** What are the needed explorations before performing a planned lumbar puncture?

- No exploration after questioning the patient about hemostasis
- Ophthalmologic exploration
- Brain imaging
- Exploration of hemostasis (prothrombin time, activated partial thromboplastin time)
- Exploration of hemostasis (prothrombin time, activated partial thromboplastin time, platelet count)

**MRQ5** What are the contraindications of a planned lumbar puncture?

- Lumbar arthrosis
- Aspirin 75 mg/day
- Intracranial hypertension with a risk of brain displacement
- Low-molecular-weight heparin (LMWH)
- Curative anticoagulation (including new anticoagulants)

**MRQ6** About lumbar puncture

- The yellow needle (20G) has a smaller diameter than the black one (22G)
- The use of Sprotte needle (atraumatic needle) is recommended
- The lumbar puncture can cause a palsy of the sixth cranial pair
- The lumbar puncture can cause an epidural hematoma
- A local anesthesia is recommended before performing a lumbar puncture

***B/ Scales evaluating the level of confidence and the level of knowledge. These scales were filled in before and after the training***

What is your level of theoretical knowledge of LP?

(Please check one numbered box in the scale below)

**
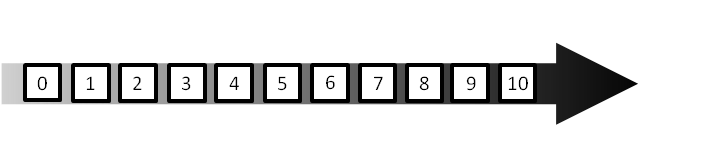
**

What is your level of confidence in the practice of LP?

(Please check one numbered box in the scale below)

**
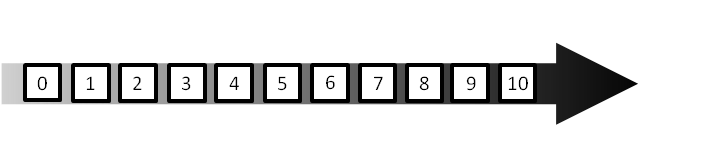
**

***C/ Evaluation of the quality of LP simulation-based training sessions by the students (QR code survey)***

| **Quality criteria** | **Mark (out of 4)** |
| --- | --- |
| Trainers' availability | 3.93 ± 0.25 |
| Quality of material | 3.64 ± 0.60 |
| Adapted to personal goals | 3.87 ± 0.33 |
| Quality of welcoming | 3.80 ± 0.46 |
| Relevance of the topic | 3.94 ± 0.23 |
| Time management | 3.76 ± 0.51 |
| Pedagogical method | 3.88 ± 0.31 |
| Theoretical presentation | 3.57 ± 0.52 |
| **Average satisfaction score** | **3.80 ± 0.14** |

***D/ Comparison of groups of students with versus without prior LP-simulation based training in the randomized study.***

|  | Total | | LP simulation group | | Control group | | p |
| --- | --- | --- | --- | --- | --- | --- | --- |
|  | n | % | n | % | n | % |  |
| **Number of students** | 41 | 100 | 27 | 66 | 14 | 34 |  |
| **Success of first LP*** | 20 | 49 | 18 | 67 | 2 | 14 | **0.0025** |
| **Need of verbal**  **or technical assistance from supervisor** |  |  |  |  |  |  | **0.017^$^** |
| Simple supervision | 7 | 17 | 6 | 22 | 1 | 7 |  |
| Verbal help | 21 | 51 | 16 | 59 | 5 | 36 |  |
| Technical assistance | 13 | 32 | 5 | 19 | 8 | 57 |  |
| **Students' subjective experience** |  |  |  |  |  |  | **0.20^#^** |
| Not comfortable at all | 1 | 2 | 0 | 0 | 1 | 7 |  |
| Quite uncomfortable | 6 | 15 | 3 | 11 | 3 | 22 |  |
| Quite comfortable | 31 | 76 | 23 | 85 | 8 | 57 |  |
| Very comfortable | 3 | 7 | 1 | 4 | 2 | 14 |  |

*PL success was defined as the sampling of CSF (at least three tubes) without technical help

^$^p value for comparison between “technical assistance” vs “simple supervision/verbal help”, Fisher’s exact test

^#^p value for comparison between “not comfortable at all/quite uncomfortable” vs “quite comfortable/very comfortable”, Fisher’s exact test
